# Marfan Syndrome Caused by a Novel *FBN1* Mutation With Associated Pigmentary Glaucoma

**DOI:** 10.1002/ajmg.a.35838

**Published:** 2013-02-26

**Authors:** John Kuchtey, Ta Chen Chang, Lampros Panagis, Rachel W Kuchtey

**Affiliations:** Vanderbilt Eye Institute, Vanderbilt UniversityNashville, Tennesse

**Keywords:** pigmentary glaucoma, Marfan syndrome, fibrillin-1, microfibril, aqueous humor outflow

## Abstract

Mutations in fibrillin-1 (*FBN1*) cause a wide spectrum of disorders, including Marfan syndrome, which have in common defects in fibrillin-1 microfibrils. Ectopia lentis and myopia are frequently observed ocular manifestations of Marfan syndrome. Glaucoma is also associated with Marfan syndrome, though the form of glaucoma has not been well-characterized. In this report, ocular examination of a patient diagnosed with Marfan syndrome based on family history and aortic dilatation was performed, including measurement of facility of aqueous humor outflow by tonography. The patient did not have ectopia lentis at the age of 42 years. Based on optic nerve appearance, reduced outflow facility, elevated IOP with open angles and clear signs of pigment dispersion, the patient was diagnosed with pigmentary glaucoma. The patient was heterozygous for a novel truncating mutation in *FBN1*, p.Leu72Ter. Histology of normal human eyes revealed abundant expression of elastic fibers and fibrillin-1 in aqueous humor outflow structures. This is the first report of a patient with Marfan syndrome that is caused by a confirmed *FBN1* mutation with associated pigmentary glaucoma. In addition to identifying a novel mutation of *FBN1* and broadening the spectrum of associated ocular phenotypes in Marfan syndrome, our findings suggest that pigmentary glaucoma may involve defects in fibrillin-1 microfibrils. © 2013 Wiley Periodicals, Inc.

## INTRODUCTION

Marfan syndrome is inherited in an autosomal dominant fashion, and caused by mutations in fibrillin-1 (*FBN1*) [Dietz et al., 1991]. The cardinal skeletal, cardiovascular and ocular features in patients with Marfan syndrome have been extensively investigated and incorporated in the revised Ghent nosology for diagnosis [Loeys et al., 2010], with significant pleiotropism and clinical variability in each system. In a large international study of more than 1,000 Marfan syndrome patients, more than half had major eye involvement, most commonly ectopia lentis (54%) and myopia (53%), with 2% of patients affected with glaucoma, although no details on the type of glaucoma were given [Faivre et al., 2007]. In a retrospective study of 573 Marfan syndrome patients examined by ophthalmologists, primary open angle glaucoma was reported as the most common form of glaucoma, with a prevalence of 2.2%, which is higher than in the general population [Izquierdo et al., 1992]. In primary open angle glaucoma, increased intraocular pressure (IOP) is caused by reduced facility of outflow of aqueous humor through the trabecular meshwork, the mechanisms of which are not well understood. We report here on a patient with Marfan syndrome caused by a novel truncating mutation in *FBN1* with associated pigmentary glaucoma, a form of open angle glaucoma accompanied by dispersion of iris pigment.

## CLINICAL REPORT

The patient was a 42-year-old Caucasian male previously diagnosed with Marfan syndrome based on family history and aortic dilatation, for which he underwent a Bentall procedure (ascending aortic graft with root replacement) in the past. In addition, he had skeletal features typical for Marfan syndrome, including joint laxity, scoliosis, and pectus deformity. He also underwent surgical intervention for pneumothorax at the age of 35 years. At presentation to our institution, his best corrected visual acuity was 20/20 in the right eye (OD) and 20/40 in the left eye (OS) with significant myopic and astigmatic correction (OD: −11.25 + 2.25 × 90; OS: −18.00 + 2.75 × 100). Central corneal thickness measured by hand-held pachymeter (Accutome, Malvern, PA) showed average thickness in each eye (543 µm). Corneal curvature and axial length measured by optical coherence biometer (IOL Master; Zeiss, Jena, Germany) revealed relatively flat corneas and long axial lengths (OD: 41.56 D/44.12 D, and 26.81 mm for corneal curvature and axial length; OS: 40.66 D/41.36 D, and 29.46 mm for corneal curvature and axial length). Dilated fundus examination showed thinning of the neuroretinal rim in the temporal aspect of optic nerve, consistent with glaucomatous optic nerve damage, more pronounced in the left eye than the right eye. Tilted optic nerve head and prominent peripapillary atrophy in each eye was also observed. There was also evidence of lattice degeneration in the peripheral retina in each eye.

**FIG. 1:**
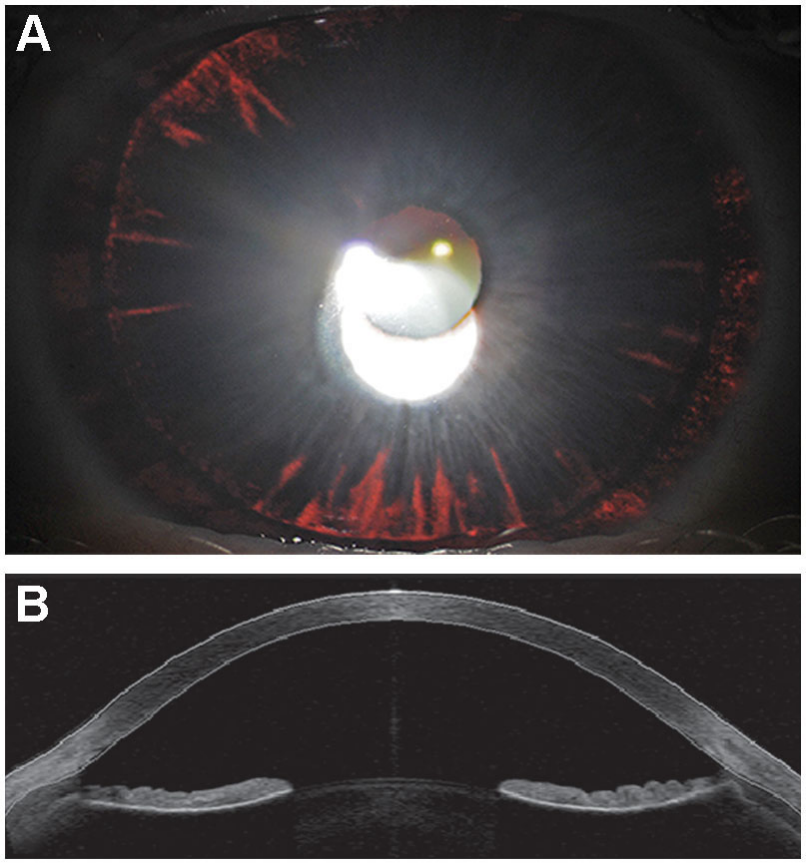
Ocular findings of a patient with Marfan syndrome. Slit lamp photo of the patient with Marfan syndrome reveals pronounced iris transillumination defects as red color (**A**). The anterior chamber angles are open and iris sustains a back-bowing configuration demonstrated by anterior segment optic coherence tomography (**B**).

Elevated IOP was first documented at 37 years of age for this patient. At presentation to our glaucoma clinic, the patient's IOP was elevated in both eyes with more pronounced elevation in the left eye (OD: 28 mmHg and OS: 43 mmHg), in the absence of IOP-lowering medications. Measurement of the facility of outflow of aqueous humor (outflow coefficient, C) with pneumatonography Model 30™ (Reichert Technologies, Depew, NY) showed significant reduction of outflow facility in the left eye (0.03 µl/min/mmHg) and minimally reduced facility in the right eye (0.37 µl/min/mmHg), consistent with the asymmetric IOP. Slit lamp examination showed characteristic findings of pigment dispersion syndrome in both eyes, including pigment accumulation on the posterior surface of the central cornea in a vertical pattern (Krukenberg's spindle), deep anterior chamber both centrally and peripherally, iris transillumination defect in a radial spoke-like pattern in the mid-periphery of the iris and a back-bowing configuration of the iris (Fig. 1A). Both lenses appeared normal in size and shape, with no indications of ectopia lentis or cataract. Gonioscopic examination showed a wide open angle with a dense and homogeneous band of dark pigment in the full circumference of the trabecular meshwork. The open angle and concave configuration of the iris was further confirmed by anterior segment optic coherence tomography (Visante OCT; Zeiss) (Fig. 1B). Based on the presence of optic nerve damage, elevated IOP with open angles and clear signs of iris pigment dispersion, the patient was diagnosed with pigmentary glaucoma.

**FIG. 2:**
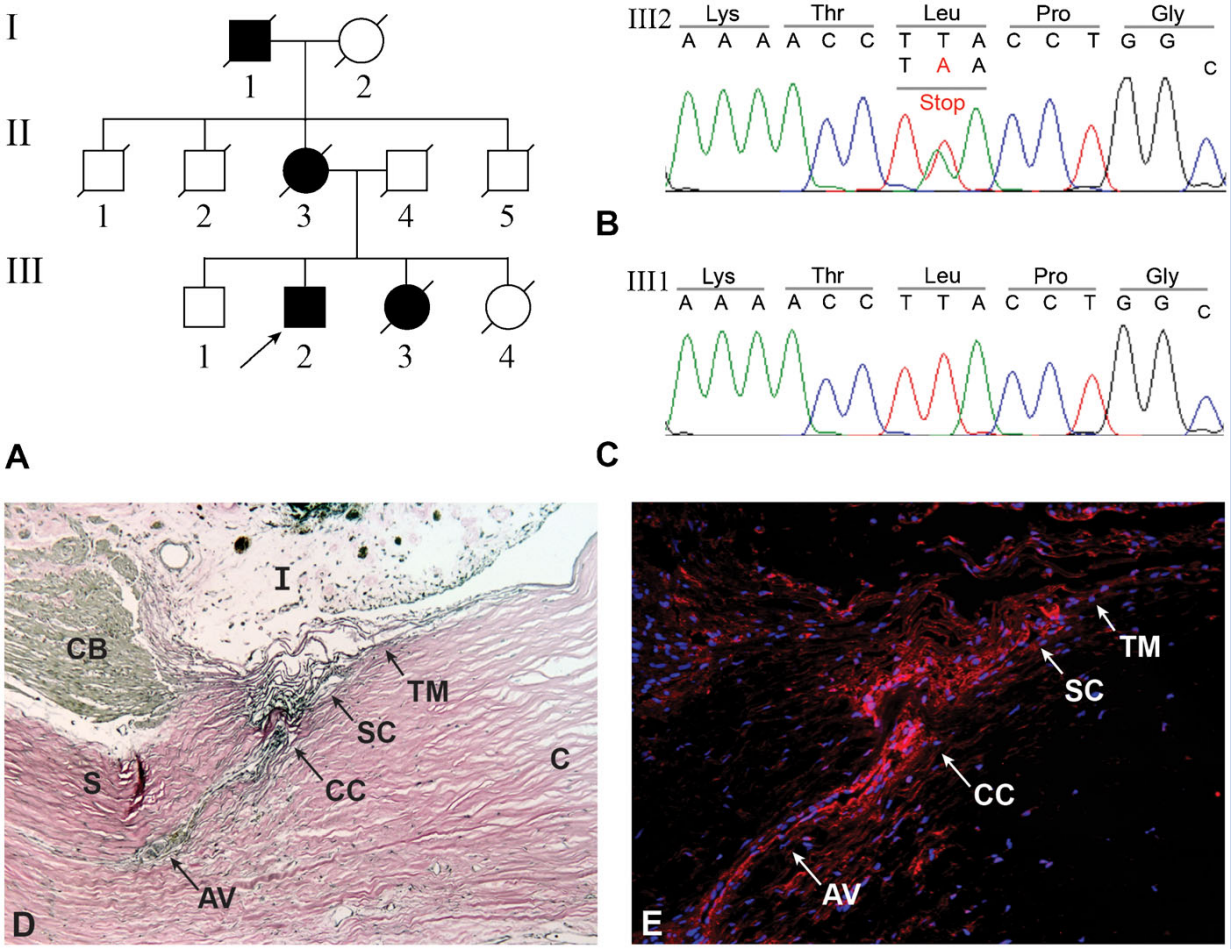
Patient pedigree, mutation screening and fibrillin-1 expression in the anterior segment of the eye. Sequencing *FBN1* revealed that the patient was heterozygous for a T > A substitution, changing a codon for leucine to a termination codon (**B**), while the patient's unaffected sibling (III1 as shown in **A**) was homozygous wild type at that position (**C**). Verhoeff Elastic–Van Gieson staining of sections from normal human cadaver eyes revealed abundant expression of elastic fibers: black staining in the aqueous humor outflow structures, including the trabecular meshwork (TM), Schlemm's canal (SC), collector channel (CC), and aqueous vein (AV), as well as in the ciliary body (CB) and iris (I), but not in the sclera (S) or cornea (C, **D**). Immunohistochemical detection of adjacent sections showed abundant expression of fibrillin-1 in aqueous humor outflow structures, including the trabecular meshwork (TM), Schlemm's canal (SC), collector channel (CC), and aqueous vein (AV) (**E**).

Autosomal dominant inheritance of Marfan syndrome in a three-generation pedigree of the patient's family is shown in Figure 2A. Although the patient had a family history of Marfan syndrome and dilated aortic root, in light of the lack of ectopia lentis, a test for *FBN1* mutation was performed. All 65 exons of *FBN1* were amplified by PCR and sequenced using an ABI3730 sequencer (Carlsbad, CA). The patient was heterozygous for a variant in exon 2 (NM_000138.4: c.221T > A) which results in a change from leucine (TTA) to a termination codon (TAA) at amino acid 74 (NP_000129: p.Leu72Ter, Fig. 2B). This variant is novel as confirmed by a search of NCBI dbSNP build 137 (Database of Single Nucleotide Polymorphisms, National Center for Biotechnology Information, National Library of Medicine; http://www.ncbi.nlm.nih.gov/SNP/) and the NHLBI Exome Variant Server (NHLBI GO Exome Sequencing Project, accessed September, 2012; http://evs.gs.washington.edu/EVS/). The patient's older brother (III1 in Fig. 2A), who has no evidence of Marfan syndrome or any ocular abnormalities did not have this mutation (Fig. 2C). Three other family members (I1, II3 and III3 in Fig. 2A) had a diagnosis of Marfan syndrome by history, but were not available for clinical examination or *FBN1* testing.

To further understand the possible mechanisms of glaucoma in this patient, we investigated expression in the aqueous humor outflow pathway of fibrillin-1 and elastic fibers, which are composed of elastin cores surrounded by fibrillin-1 microfibrils. Histology using Verhoeff Elastic–Van Gieson stain as described before [Hann and Fautsch, 2011] revealed abundant elastic fibers in the aqueous humor outflow pathway structures and the stroma and epithelia of the iris as well as the ciliary body (Fig. 2D, black staining). Detection of fibrillin-1 (anti Fibrillin-1; EPC, Owensville, MO) by immunofluorescence on adjacent sections showed intense staining of the aqueous humor outflow pathway structures (Fig. 2E), consistent with previous studies [Wheatley et al., 1995]. The location of elastic fibers and fibrillin-1 expression suggest that mutations in *FBN1* could directly affect the aqueous humor outflow pathway.

## DISCUSSION

The precise mechanisms for the development of pigmentary glaucoma remain unclear. Pigment dispersion from the iris epithelium is likely due to the rubbing of the lens zonules against the posterior surface of the iris when the iris sustains a backward bowing configuration, as proposed over three decades ago [Campbell, 1979] and supported by the clinical observation that the iris transillumination defects spatially coincide with lens zonules. The cause of the backward bowing configuration of the iris is less clear. The concept of reverse pupillary block offers an explanation in which aqueous humor trapped in the anterior chamber causes a pressure differential which forces the backward bowing of the iris. Releasing trapped aqueous humor by laser peripheral iridotomy has been proposed as a treatment [Karickhoff, 1992], however, inconsistent results have been reported [Potash et al., 1994; Scott et al., 2011]. The backward bowing of the iris has been proposed to be caused by intrinsic iris defects [Kupfer et al., 1975], which would be consistent with the *FBN1* mutation and presence of elastic fibers and fibrilin-1 expression in the iris observed in this study. The *FBN1* mutation in this patient could cause backward bowing by compromising the mechanical properties of the iris.

The cause of increased IOP in pigmentary glaucoma is also not clear and may not be a simple matter of dispersed iris pigment obstructing the aqueous humor outflow pathway. Although one study conducted by Grant [1963] demonstrated that pigment granules perfused in human autopsy eyes reduced aqueous humor outflow facility, perfusion of living monkey eyes with uveal pigment particles caused only a transient reduction of outflow facility [Epstein et al., 1986]. In addition, histological studies of human eyes with pigmentary glaucoma showed that the amount and location of accumulated pigment in the trabecular meshwork were not sufficient to account for increased resistance to aqueous humor outflow [Murphy et al., 1992]. Another possible explanation for reduced aqueous humor outflow is that trabecular meshwork cells become overloaded with phagocytosed material and are removed by macrophages, resulting in an acellular and collapsed trabecular meshwork structure [Gottanka et al., 2006].

Independent of iris pigment dispersion, an intrinsic defect in aqueous humor outflow caused by *FBN1* mutations is certainly possible since our histological study and others [Wheatley et al., 1995; Hann and Fautsch, 2011] have shown fibrillin-1 expression in the outflow pathway. The abundance and expression pattern of fibrillin-1 microfibrils suggest an important functional role for these structures in normal aqueous humor outflow, which may be impacted by mutations in *FBN1*.

An intrinsic defect in the outflow pathway due to *FBN1* mutations would be consistent with several long-standing observations in glaucoma research. Fibrillin-1 is a major component of microfibrils, which provide stretchable support in tissues such as blood vessels and skin and play a central coordinating role in signaling through transforming growth factor beta (TGFβ). Microfibril defects could account for several proposed mechanisms for reduced outflow facility in glaucoma, such as the accumulation of sheath-derived plaques derived from degraded elastic fibers observed in glaucomatous trabecular meshwork [Lutjen-Drecoll et al., 1986], and reduced pulsatile aqueous humor outflow in glaucoma patients [Johnstone et al., 2011]. In addition, since they form a major reservoir for latent TGFβ, defects in microfibrils could provide a mechanistic explanation for the long-standing observation that TGFβ concentration is elevated in the aqueous humor of glaucoma patients [Fuchshofer and Tamm, 2012].

Although there has been one previous report of a Marfan syndrome patient with pigmentary glaucoma [Doyle et al., 2005], this is the first report of pigmentary glaucoma in a patient with a confirmed *FBN1* mutation. Our report strengthens the association of pigmentary glaucoma with Marfan syndrome that is caused by fibrillin-1 mutation. However, the inability to perform ocular examination on other affected family members limits the establishment of a causal relationship between pigmentary glaucoma and the specific *FBN1* mutation identified in our patient. Our patient did not have ectopia lentis at the age of 42 years. Lack of such ocular phenotype in our patient is consistent with the previous report that ectopia lentis phenotype was commonly seen in patients who had *FBN1* missense mutations either substituting or producing a cysteine residue [Faivre et al., 2007]. In addition to identifying a novel mutation and broadening the spectrum of ocular phenotypes in Marfan syndrome, our findings suggest that pigmentary glaucoma may be another disease caused by mutations in *FBN1*. The abundant expression of fibrillin-1 protein in the iris and aqueous humor outflow pathway offer mechanistic explanations for both the unusual backward bowing configuration of the iris and a possible component of reduced outflow facility.

It has long been appreciated that *FBN1* mutations are associated with vastly different phenotypes ranging from Marfan syndrome and Weil–Marchesani syndrome to isolated ectopia lentis. The *FBN1* mutation found in this study is a truncation mutation, which usually is associated with more severe Marfan syndrome phenotypes. Our findings suggest that mutations in *FBN1* and other genes involved in microfibril structure and function may be found in patients with pigmentary glaucoma and possibly other forms of glaucoma.
